# Telehealth and digital health innovations: A mixed landscape of access

**DOI:** 10.1371/journal.pdig.0000401

**Published:** 2023-12-15

**Authors:** Jimmy Phuong, Patricia Ordóñez, Jerry Cao, Mira Moukheiber, Lama Moukheiber, Anat Caspi, Bonnielin K. Swenor, David Kojo N. Naawu, Jennifer Mankoff

**Affiliations:** 1 UW Medicine Research Information Technologies, University of Washington, Seattle, Washington, United States of America; 2 Department of Information Systems, University of Maryland Baltimore County, Baltimore, Maryland, United States of America; 3 Paul G. Allen School of Computer Science, University of Washington, Seattle, Washington, United States of America; 4 The Picower Institute for Learning and Memory, Massachusetts Institute of Technology, Cambridge, Massachusetts, United States of America; 5 Institute for Medical Engineering and Science, Massachusetts Institute of Technology, Cambridge, Massachusetts, United States of America; 6 Taskar Center for Accessible Technology, Seattle, Washington, United States of America; 7 Johns Hopkins Disability Health Research Center, Baltimore, Maryland, United States of America; 8 Johns Hopkins School of Nursing, Baltimore, Maryland, United States of America; 9 Wilmer Eye Institute, Johns Hopkins School of Medicine, Baltimore, Maryland, United States of America; 10 Department of Epidemiology, Johns Hopkins Bloomberg School of Public Health, Baltimore, Maryland, United States of America; 11 Meharry Medical College School of Medicine, Nashville, Tennessee, United States of America; Kamuzu University of Health Sciences: University of Malawi College of Medicine, MALAWI

## Abstract

In the wake of emergent natural and anthropogenic disasters, telehealth presents opportunities to improve access to healthcare when physical access is not possible. Yet, since the beginning of the COVID pandemic, lessons learned reveal that various populations in the United States do not or cannot adopt telehealth due to inequitable access. We explored the Digital Determinants of Health (DDoHs) for telehealth, characterizing the role of accessibility, broadband connectivity and electrical grids, and patient intersectionality. In addition to its role as an existing Social Determinant of Health, Policies and Laws directly and indirectly affect these DDoHs, making access more complex for marginalized populations. Digital systems lack the flexibility, accessibility, and usability to inclusively provide the essential services patients need in telehealth. We propose the following recommendations: (1) design technology and systems using accessibility and value sensitive design principles; (2) support a range of technologies and settings; (3) support multiple and diverse users; and (4) support clear paths for repair when technical systems fail to meet users’ needs. Addressing these requires change not only from providers but also from the institutions providing these systems.

## Introduction to telehealth

Telehealth, the modalities for remote didactic communication and healthcare access with healthcare providers [1], is undergoing a revolution to improve its reach and utility in healthcare. Promoted from the Affordable Care Act of 2008, telehealth is an umbrella term referring to analog and audio-video out-of-office visits as alternatives to in-person healthcare [1,2]. Telehealth includes Telemedicine and a variety of non-physician services (e.g., telenursing, telepharmacy, and linguistic interpretation) and can be discussed synonymously with integrated remote care modalities, such as mobile health and E-health platforms [1].

The need and implementation of telehealth services escalated with the Coronavirus Disease 2019 (COVID-19) pandemic [2–5], yet the results thus far illustrated both its potential to radically increase healthcare access and how it can disenfranchise certain groups. Apart from analog telecommunication, telehealth relies on remote meeting software and broadband infrastructure to facilitate communication [2,3]. Areas with unstable, unreliable electric and telecommunication services would be prone to connectivity issues. Overtime, repeated lag in device ownership and innovation diffusion contributes to the cultural digital divide, though these disproportionate health disparities originate from infrastructural factors and the lack of equitable tools designed for patients of diverse needs and care team settings [4].

In the following sections, we discuss the benefits and risks of telehealth, highlighting structural and intersectional factors that contribute to telehealth adoption. We aimed to understand the landscape of telehealth implementation, considerations for accessibility design to include patients with different functional disabilities, and the relationships with critical concepts such as digital divide and Social Determinants of Health (SDoHs).

### The benefits of telehealth

While telehealth has many purported benefits, we highlight 3 major thematic advantages for telehealth. First, telehealth can improve healthcare access and reach to those who otherwise live in “healthcare deserts,” where in-person access to care requires physical travel and time expenditures that may be economically prohibitive [4,5]. Second, telehealth can improve healthcare access options for people with disabilities by reducing travel for medical visits [6,7]. Approximate 27% of US adults have a functional disability and face barriers accessing healthcare [8]. Telehealth circumvents mobility barriers related to travel-time and wait-times, allowing for improved engagement for healthcare [2]. Third, telehealth video conferencing may mask in-person characteristics that invoke provider bias. For example, people of over-average weight reported experiencing discriminating biases during healthcare encounters, though this phenomena may be shared and intersectional among multiple subgroup identities [9]. Over video, a healthcare provider may be less likely to respond negatively to certain identity characteristics.

### The risks of telehealth

In contrast, telehealth may increase the risk of unequal access to care as it may ossify existing disparities [2,7]. Those who reside in “healthcare deserts” and experience unreliable broadband connectivity may not be able to access telehealth as an alternative to in-person healthcare. Healthcare access can be made worse when certain healthcare services with staff shortages shift towards online instead of in-person care, a concern with mental healthcare and counseling [10,11]. Moreover, telehealth systems may be predominantly designed for subpopulations who experience few barriers to adoption, excluding the needs of those who lack technology literacy, have unmet visual accessibility needs, or do not have the financial capabilities to pay for services [4,7,12].

Gradually, telehealth may incorporate complementary technologies, supporting measurements and diagnostic data collection at-home [6,7,13]. The lack of complementary and accessible in-home technology support may limit the benefits of healthcare services available. Even if a video call is accessible to a blind person, the telehealth experience may be limited if there are no accessible option to take measurements. However, paired technologies may fail to account for diverse users, such as a wheelchair user whose low step count does not reflect their actual activity level. These systems must be compliant with the Americans with Disabilities Act of 1990 (ADA) guidelines, not only for default settings but also settings allowing for personalization [14].

Telehealth may obscure or enhance visible presentations [15]. Video conferencing may offer a deeper window into the patient’s home environment and accidentally disclose private information more than the patient would have desired. Alternatively, visibility of symptoms and functional disabilities may be obscured, which can influence provider diagnostic coding and response for undiagnosed disabilities. A person with a chronic illness may present fatigued in-person, but video conference may obscure the fatigue, leading to the provider not taking symptoms seriously, which increases likelihood for misdiagnosis or delayed actions. Increased risk of severe COVID-19 and hospitalization actions varies depending on types of functional disabilities [16], in which visibility of symptoms and presentations can present a challenge in patient–provider decision-making.

## Digital determinants of health

Akin to SDoHs, Digital Determinants of Health (DDoHs) highlight contemporary constructs about digital health innovations with significance towards healthcare equity. DDoHs highlight conceptually distinct facilitators and barriers from SDoHs for the diffusion of medical and public health digital innovation into the general populace, or the lack thereof when considering the effects of a digital divide.

Pre-pandemic, the World Health Organization defined Digital Literacy as “literacy in information and communication technologies and access to equipment, broadband and the internet” [17]. Since the onset of the COVID-19 pandemic, telehealth played a key role for emergency triage, access to medical professionals, medical follow-up, and the processes for referrals to in-person care, while abiding with social distancing recommendations [18]. Yet, the pandemic has also demonstrated how telehealth may not be a pragmatic option for certain patients or providers, serving as an example for implementing and adoption of many other digital innovations. While digital literacy may explain some of the nuances facilitating or barring telehealth technological adoption, recent studies have identified critical drivers of digital divide, factors which we refer to as DDoHs [4,17]. These DDoHs can be observed with telehealth adoption to further increase disparities in access to healthcare in marginalized communities.

**Fig 1:**
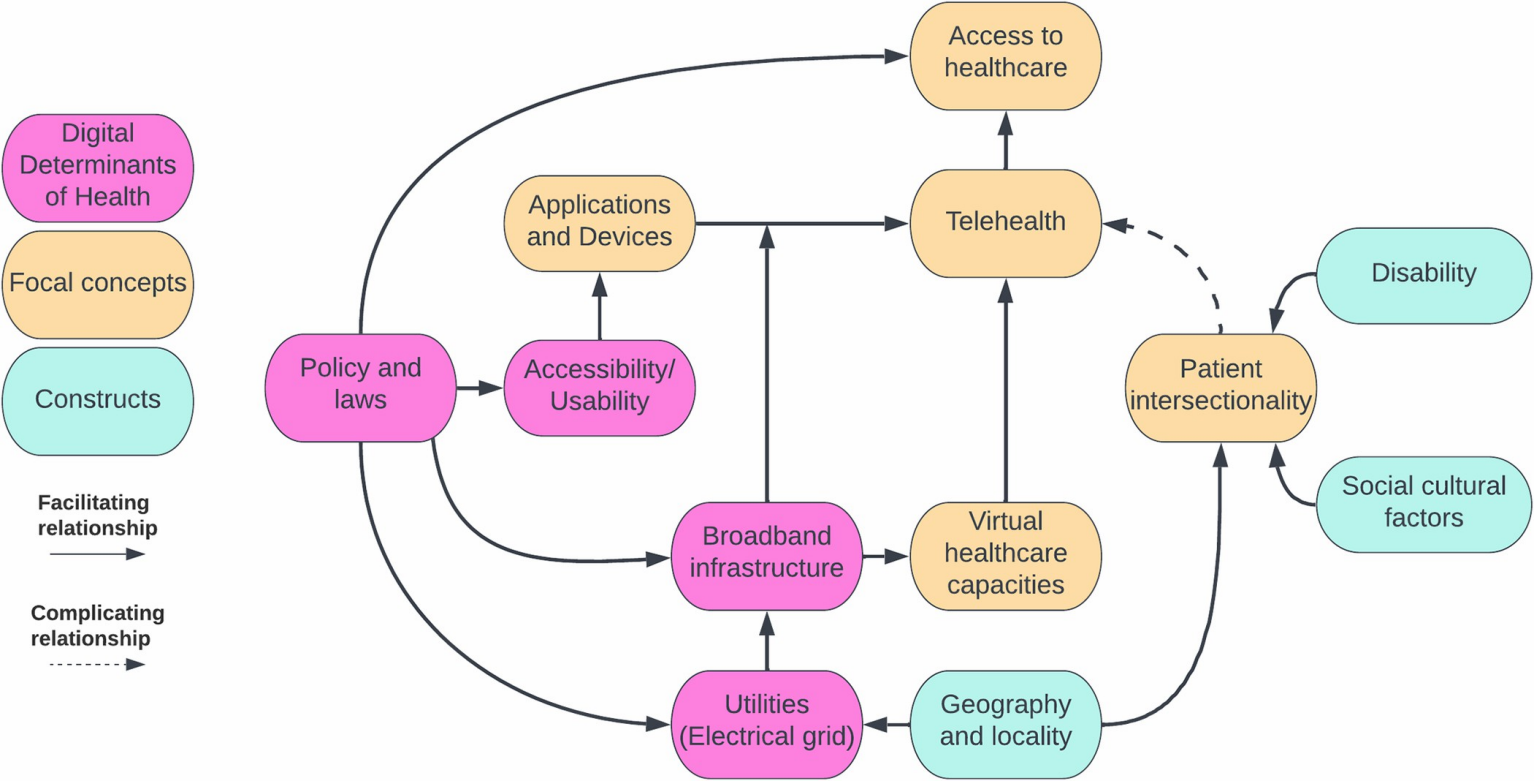
Simplified conceptual relationships between DDoHs and telehealth. “Policies and laws” are SDoHs for access to telehealth care. “Policies and laws” intervene with the status quo and influence DDoHs for telehealth care; therefore, it can be considered a DDoH of telehealth.

The ability for healthcare systems to adopt telehealth is contingent on a myriad of factors (Fig 1) [1,19,20], including broadband infrastructure and its reliance on the electrical grid. In that sense, the landscape geography and localities with stable broadband infrastructure can explain the resources available to key actors. Yet, for patients who experienced the intersection of unmet social needs, resource barriers, and different forms of discrimination, experiences with inequity can reduce ones’ willingness to engage in such technologies. Principles of Accessibility facilitate reach to marginalized segments of the population who experience diverse variations in human ability [3,14]. Value sensitive design is one such approach for designing solutions that incorporate the stakeholders, identifying values alignments and value conflicts, and envisioned scenarios within the design process [21]. We note that “Policy and Laws” can directly legislate access to care as a healthcare options, but it can also be viewed as an indirect influence upon telehealth by way of the digital determinants of health (i.e., usability and accessibility design, broadband infrastructure investments, and maintenance of public utilities). In the following sections, we elaborate on how these DDoHs influence telehealth.

### Broadband infrastructure

For adoption of telehealth and other digital innovations to be facilitated, patients, healthcare providers, and health systems must adapt to adopt these technologies as part of their healthcare workflows [19,22]. Access to many digital health innovations is contingent on access to hardware components and reliable broadband connectivity [4,22–25]. Analog options may be preferred as feasible methods for continuation of core services in lieu of reliable broadband connectivity infrastructure or its dependence on the electrical grid [26]. In more rural areas, broadband internet may be unaffordable even if it were regionally available, leaving people behind in technological literacy and the digital divide [4,25]. Broadband infrastructure requires investments to maintain and update hardware and security, data access contracts, and various other costs felt by individuals and regional governance. Due to distance to care and unresolved internet connectivity, telehealth services may continue to be out of reach for many minority, rural, and low socioeconomic communities from telehealth services.

Reports indicate that telehealth adoption in response to the COVID-19 public health emergency has met various social, practical, and accessibility of implementation challenges [2,5,18,23]. Pilot implementations of telehealth found that providing tablets alone could not overcome connection rate hurdles, requiring in-home hardware installations to strengthen broadband connectivity [1,2,22]. The highest rate of adoption were among Medicare and Medicaid recipients [2]. Specifically, telehealth enables continued access to care for most but has done little for the uninsured young adult populations who experience barriers in access to care and the highest rates of emotional distress from COVID-19 [2,27].

### Accessible electrical grid after natural disasters

Natural disaster events destabilize and deteriorate electrical grid infrastructure and services that depend on them [28,29]. Climate-related disruptions to the electrical grids in California and Texas put patients who were reliant on electrical life support at risk [24–26]. Unreliable electrical sources can impact medication refrigeration and complicate chronic care management. In Puerto Rico, Hurricanes Irma and María in 2017 wiped out the electrical grid for over a year for the majority of the island, creating the longest blackout in the history of the Americas [30]. Delays in action to modernize the electrical grid allowed power outages to continue weekly across the island, leaving Puerto Ricans juggling with challenges at work, at school, and in managing health and wellness [30]. Downed electrical grid can have a direct impact on care management and disrupt access to telehealth and emergency response for broad geographic areas.

With modernization of the electrical grid and broadband infrastructure, increased availability may not translate to increased accessibility. Where critical infrastructure is unreliable or not cost-effective, it can be a source of digital health inequity. For some, newly built electrical grids may be available but financially unaffordable except to private entities and commercial services [4,31]. Mountainous terrains or flood-prone areas are not conducive to building sustainable infrastructure and often get low priority for development. In such circumstances, reliance on analog options persists and adoption of telehealth, other digital innovations, and technological literacy falters.

## Capacity building for telehealth: A systems engineering approach

**Fig 2:**
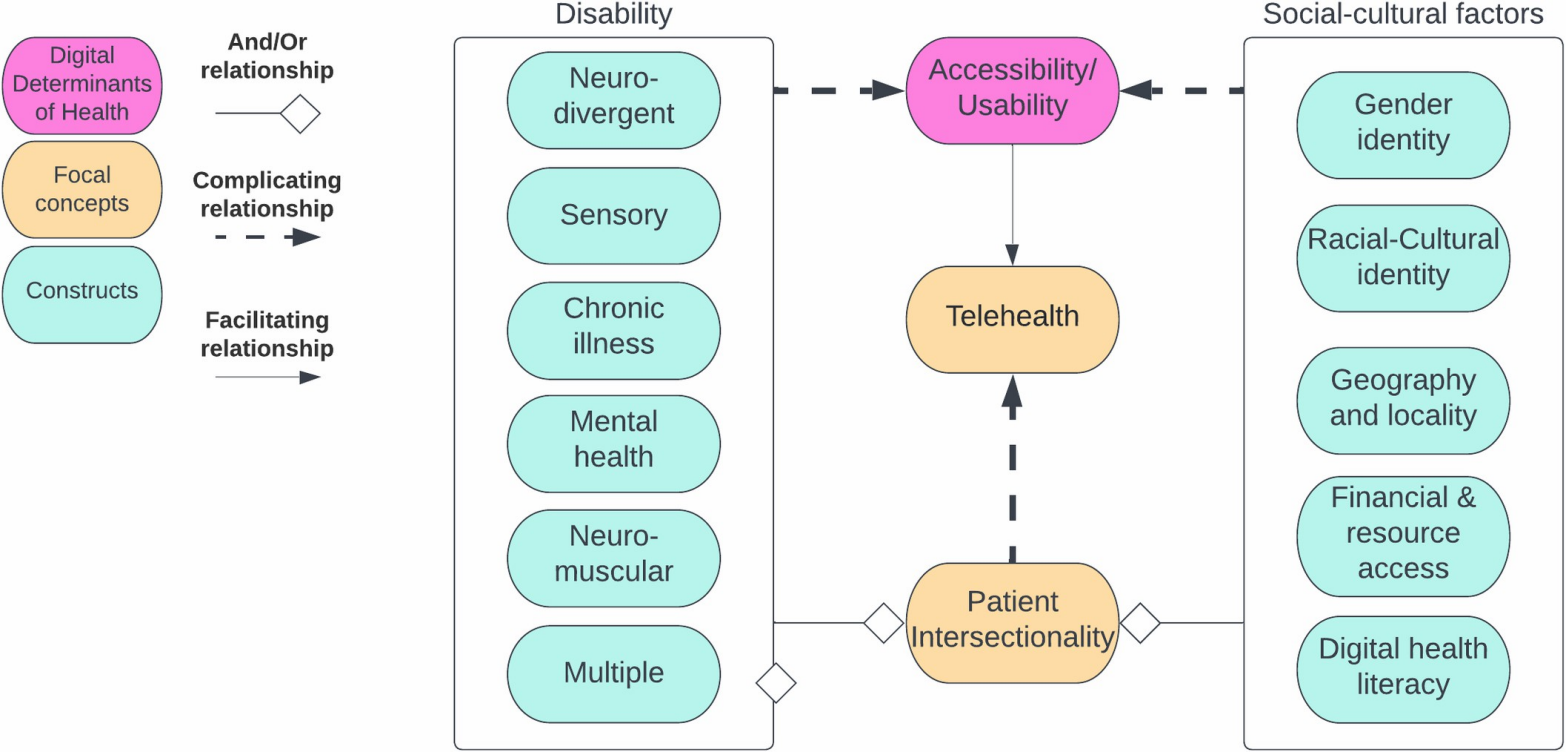
Factors towards patient intersectionality and towards telehealth accessible design.

Medical systems are already complex, involving a highly connected system of people, resources, processes, and institutions. Telehealth is an attempt to improve care, but also involves disruption to the existing systems [18,19,23], with the potential for wide-ranging positive and negative consequences. As an improvement methodology, we should build capacity through every discrete part of the system (i.e., people, resources, processes, and institutionally) to properly equip and better manage both complexity and risk, understanding and accommodating patient needs for healthcare accessibility and understanding patient intersections (Fig 2). Patients may have one or more functional disabilities, which can change their healthcare utilization over time. Social risk factors are intersectional and dynamic, changing what tools and resources are needed as one progresses in life and whether those needs are responsively and equitably met, meriting that social risk factors and functional abilities/disabilities are supportively inquired. Taking a holistic engineering systems approach may be beneficial, to avoid focusing on discrete interventions whose effects are narrowly monitored, particularly when they are used to address the needs of small subpopulations. Engineers have long understood that complex problems require a systems view and that attempts to make things better can themselves introduce new risks into a system. In the paragraphs below, we take an engineering systems approach to identify the capacity-building requirements needed to allow telehealth to improve healthcare for all patients.

### Barriers in the patient–provider clinical workflow

Telehealth interactions, and health interactions in general, increasingly depend on technology to support all aspects of the process, from communicating with doctors to scheduling appointments and everything in between. Digital systems do not only impact the telehealth experience; there is significant overlap in all efforts before and after telehealth and in-person visits. However, digital systems, such as electronic health records and wearable monitoring devices, are used even during in-person appointments. They have become so ubiquitous as to frequently be unavoidable requirements for access to care. The United States has observed a shortage of mental health provider, where COVID has seen mental healthcare visits shift overwhelmingly towards telehealth visits [10,11,32]. Wherever possible, the shift towards telehealth can reach more patients, but the change makes digital literacy a requirement to receive care.

Throughout this transition towards telehealth, digital literacy and understanding about security and privacy are assumed. Patients often have to learn to interact with entirely new systems via email notifications, sometimes a different system for each clinician they see. In a study that compared healthcare experiences of those with and without disabilities, individuals with disabilities encountered unique challenges that were specific to their disability [33]. While not all participants in the study reported a lack of standard of care, some participants reported on how they felt that their symptoms were interrelated and that no one physician could treat all of their symptoms [33]. Despite the use of electronic health records (EHRs), patients felt that inter-provider communication was poor, even among those within the same network [33]. Further, many of these systems are inaccessible to people with disabilities, and disability-specific systems may not be familiar to clinicians [34]. Consent becomes perfunctory, less meaningful for every new account with a new software application downloaded. Patients rarely have control over these aspects of patient experience, and a paucity of these experiences are documented.

### Unfamiliar and inaccessible digital systems

We defined digital systems to be inclusive of, but are not limited to, EHRs, patient health records, telehealth services, and wearable devices like glucose monitors. Most applications of telehealth now use custom, siloed messaging systems that protect privacy by keeping all communication within the system, though data retention policies may hinder patient access to information and private correspondences [35]. A patient may be required to log into a myriad of different websites, often with little guidance to establish and maintain these accounts. The awareness and preservation of documents related to these separate systems can be imperative for reporting legal discourse, reimbursement, determining coverage, and continued access to services. This can be further compounded by state-to-state legal requirements around patients’ rights, such as who can see an adolescent’s medical records.

Telehealth may reduce visibility or presentation of symptom severity over a video camera than in-person. The visibility affects provider belief in what patients self-report and, ultimately, on what gets actioned upon [36]. It is a matter of digital literacy to make sure that the systems do not expose too much and that the patients can retain the information they need.

While automation is typically viewed as a positive, automation that has built assumptions about “normal” abilities further perpetuates ableism [37]. For example, facial recognition software are increasingly used in medical care settings, though with less success among older adults with dementia [38]. Proprietary algorithms may be protected by intellectual property laws, obfuscating the reasoning for automated responses. Where automated algorithms are used to make determinations, patients and providers may have no recourse to understand or change the output.

These custom systems are rarely designed using Principles of Accessibility to accommodate the range of user needs. Even for someone with high digital literacy, they can be challenging to learn and navigate [7,39]. The system may be inaccessible for someone who only has a tablet or phone, not a desktop computer [6]. Similarly, support for screen reader use may be entirely lacking [ibid.]. Recent changes in California’s automating billing procedures for In-Home Supported Services require navigating inaccessible phone or online AI verification procedures, which can have built-in assumptions about how fast someone can respond, what accent they have, or other biometric characteristics [7,37,40]. Without direct testing with disabled users, the generated system may be compliant with web accessibility guidelines and laws but may be essentially unusable.

### Impact of digital technologies on consent

The increasingly digital nature of the telehealth (and health) experience impacts the consent process. Society as a whole has abdicated the consent process to lawyers as the number of lines of text and technical jargon one must read to consent to digital life properly has exceeded what is reasonable [41–43]. Whether in-person or digitally signed, it is a common experience to skip over consent details, undermining the importance, visibility, and patient literacy for a meaningful consenting process [15,41,43].

Similarly, in telehealth, patients often treat consent as something to get past—a long scrolling window of text is rarely read but just signed. Consent is elided by the temporal gap between when a system is implemented and when it is used. Thus, if a programmer encodes gender as a binary or formats a hospital bracelet to prominently show a patient’s legal name even when they have communicated their consent and preferences, the programmed encodings are structurally embedded.

### Complementary technologies

We often consider telehealth as a live video chat experience, perhaps complemented by interactive EHR systems such as MyChart. However, in practice, telehealth is gradually incorporating mobile, virtual, and wearable devices and sensors (e.g., vital signs, physical activity), continuous glucose monitors (e.g., blood insulin levels), and other technologies to support chronic care management and integrative therapies [13,23]. Sometimes, these are used by patients alone, sometimes reported verbally or digitally to healthcare providers, and sometimes to third parties such as when third-party surveillance is required for insurance to pay for a technology.

While telehealth inequities may exist for people with disabilities, there are critical data gaps that limit our ability to identify and address these disparities. Currently, disability status is not routinely collected as a core demographic element in EHRs, although some studies sought to understand the gaps in healthcare processes. This lack of information makes it impossible to either address telehealth gaps for people with disabilities or to meet the healthcare accessibility needs of patients [1,7,44,45].

Home medical test usability are perhaps the most recent publicly discussed example of inaccessible home telehealth technologies [7]. Physical interfaces and apps provided with these technologies may be partly or wholly accessible to people with disabilities. In addition, they may encode hidden assumptions, such as not allowing for certain heights above a certain age, excluding people with dwarfism [46]. They demarcate “what it means to be a legible human and whose bodies, actions, and lives fall outside… [and] remapping and calcifying the boundaries of inclusion and marginalization [37]. Again, these decisions are often made by programmers and challenging to change.

Home monitoring apps and devices such as Fitbit, Strava, and Continuous Glucose Monitors are another important example of inaccessible telehealth technologies. For example, Fitbits may not count exercise in a wheelchair as fitness time [46], encoding activity assumptions about what behaviors count. Furthermore, when digital health technologies are introduced, they often fail to reach the audience who would benefit from them the most due to limited awareness, resource or infrastructure constraints to support the technologies, and perceived benefits to adopt and continue use.

Surveillance is often built into these technologies. Automated tracking of use has been deployed with continuous positive airway pressure (CPAP) machines [47] and to track the use of prosthetic legs [37]. Such surveillance is used to design who is “compliant enough” to deserve continued device use, but the information collected lacks nuance of the circumstances and may encode infrastructural biases [40]. These systems may make assumptions about reliable network connectivity or that a person does not have interacting disabilities.

On a positive note, the ability to create custom solutions for health monitoring and in-home treatments are increasing with the availability of smartphones. Smartphone applications are now a powerful tool that can noninvasively monitor important vital signs, including respiration rate, blood oxygenation, variations in blood pressure, or medical conditions such as anemia, jaundice, or sleep apnea. With ease in collecting this health information, patients can better personalize their care with their healthcare providers. The advent of 3D printing has also allowed for custom attachments to the phone that opens the door to other screening and treatment processes [48–50]. A study has shown the feasibility of using a 3D-printed attachment to the smartphone to perform blood clot testing [49]. However, these technologies still must consider accessibility and control for people with disabilities and chronic health conditions. Given the long history of in-home health hacks that are part of disability communities [7,51], it should be a given that the specification of such technologies are supported not just for but by patients themselves.

## Conclusions

To summarize, digital systems lack the flexibility, accessibility, and usability to inclusively provide the essential services patients need. This impacts not only access but also quality of care and consent. Addressing this requires change not only from providers but also from the companies providing these systems. We propose the following recommendations:

1. Design technology and systems using accessibility and value sensitive design principles

User interfaces must be intuitive, prioritizing understandability of icons and text, as well as streamlined navigation. Further, they must support best practices for user-centered design to reduce overall physical and cognitive burden and increase understandability to those with reduced digital literacy. This is especially important for consent, which must be accessible and visible to patients to be compliant with ADA guidelines [7]. They must consistently implement standards and plug-in solutions to enable sign language, closed captioning, or the appropriate interpretation on-screen as the services being provided, even for unscheduled appointments.

Usability design approaches, such as value sensitive design [21], require an iterative process that is responsive to users. This includes developing methods to capture user feedback from patients and providers, then creating systems to improve the technology based on this feedback.

2. Support a range of technologies and settings

We must support compatibility with mobile devices and tablets so that those without access to desktops and laptops can still use services. Moreover, we must support compatibility with external assistive technology and accessibility features native to all operating systems, such as sensing technologies and other complementary technologies. They must support multiple available modes of communication to allow patients to select the mode that is most accessible to them (e.g., ability to send voice-based messages through patient portals and text-based messages during a telemedicine encounter). Finally, applications of privacy preserving technologies, such as video background blurring and access via headphones, would preserve patient privacy and enable alternative options for use across the range of realistic settings.

3. Support multiple and diverse users

Full support for diverse users requires enabling multiple users of the same account through proxy status as well as the ability for multiple individuals to join a telemedicine encounter, if more than one type of assistance is required, like with a qualified sign language interpreter and a family member. Similarly, it is important to support preferred names and pronouns. Support for this must extend beyond the technology. Providers–organizations must train for providers to help them to overcome biases that are more likely to be expressed in telehealth settings.

4. Support clear paths for repair when technical systems fail to meet user needs

We must collect data to determine the impact of these systems and technology, including for groups that experience healthcare inequities. Further, we must provide transparency and control over IT decisions and algorithms, including support for preferred names and pronouns, interpretable machine learning, information about why decisions are made, and legal recourse for understanding and modifying decisions.

This burden should not solely be on the shoulders of users. We must create systems of accountability to ensure that the data make actual changes to technology and close gaps for oppressed/marginalized groups. We must use the information learned from this process to educate and enhance the next generation of developers/designers and technologies.

Policies and laws have been identified as a social detriment of health and have the potential to address the determinants to digital health. Policies and laws can create pathways for people from vulnerable populations whose healthcare would be significantly improved through telehealth. Thus, policies and laws are both a social and digital determinant to health.
